# Detection of Disease-Causing SNVs/Indels and CNVs in Single Test Based on Whole Exome Sequencing: A Retrospective Case Study in Epileptic Encephalopathies

**DOI:** 10.3389/fped.2021.635703

**Published:** 2021-05-13

**Authors:** Dan Sun, Yan Liu, Wei Cai, Jiehui Ma, Kun Ni, Ming Chen, Cheng Wang, Yongchu Liu, Yuanyuan Zhu, Zhisheng Liu, Feng Zhu

**Affiliations:** ^1^Department of Neurology, Wuhan Children's Hospital, Tongji Medical College, Huazhong University of Science and Technology, Wuhan, China; ^2^Department of Pediatrics, Tongji Hospital, Tongji Medical College, Huazhong University of Science and Technology, Wuhan, China; ^3^Department of Hematology, Wuhan Children's Hospital, Tongji Medical College, Huazhong University of Science and Technology, Wuhan, China; ^4^Xiangyang Central Hospital, Affiliated Hospital of Hubei University of Arts and Science, Xiangyang, China; ^5^Department of Cardiology, Union Hospital, Tongji Medical College, Huazhong University of Science and Technology, Wuhan, China; ^6^Aegicare Technology Co., Ltd. Shenzhen, China; ^7^Clinic Center of Human Gene Research, Union Hospital, Tongji Medical College, Huazhong University of Science and Technology, Wuhan, China

**Keywords:** epileptic encephalopathies, whole exome sequencing, genetics, copy number variation, variant

## Abstract

**Background:** Epileptic encephalopathies (EEs) are a pediatric entity with highly phenotypic and genetic heterogeneity. Both single nucleotide variants (SNVs)/Indels and copy number variations (CNVs) could be the causes. Whole exome sequencing (WES) is widely applied to detect SNVs/Indels, but the bioinformatics approach for detecting CNVs is still limited and weak. In the current study, the possibility of profiling both disease-causing SNVs/Indels and CNVs in a single test based on WES in EEs was evaluated.

**Methods:** The infants diagnosed with EEs were enrolled from a single pediatric epilepsy center between January 2018 and February 2020. Demographic and clinical data were collected. In WES data, the pathogenic SNVs were identified through an in-house pipeline, and pathogenic CNVs were identified by CNVkit. The diagnostic rate was evaluated, and the molecular findings were characterized.

**Results:** A total of 73 infants were included; 36 (49.32%) of them were males. The median age was 7 months. Thirty-two (43.84%) infants had been diagnosed with epilepsy syndrome. The most common type of syndrome was West syndrome (22/73, 30.1%), followed by Dravet syndrome (20/77, 27.4%). Fifty-four (73.97%) had intellectual development delay. The genetic cause of EEs, pathogenic or likely pathogenic variants, were successfully discovered in 46.6% (34/73) of the infants, and 29 (39.7%) infants carried SNVs/Indels, while 5 (6.8%) carried CNVs. The majority of the disease-causing variants were inherited in *de novo* pattern (25, 71.4%). In addition to showing that the variants in the ion channel encoding genes accounted for the main etiology, we discovered and confirmed two new disease-causing genes, *CACNA1E* and *WDR26*. Five discovered CNVs were deletions of 2q24.3, 1p36, 15q11-q13, 16p11.2, and 17p13.3, and all were confirmed by array comparative genomic hybridization.

**Conclusion:** The application of both SNVs/Indels and CNVs detection in a single test based on WES yielded a high diagnosis rate in EEs. WES may serve as a first-tier test with cost-effective benefit in EEs.

## Introduction

Epileptic encephalopathies (EEs) are characterized by frequent severe seizures, severe electroencephalography (EEG) abnormalities, and intellectual/developmental disabilities (1–3). EEs commonly occur during infancy and early childhood, with poor clinical outcome. They are highly heterogeneous in clinical features, including West syndrome (WS), Dravet syndrome (DS), Ohtahara syndrome (OS), migrating partial epilepsy of infancy (MPEI), and other related epilepsy syndromes (4–6). A range of etiologic spectra in epilepsy has been recognized, including structural, genetic, infectious, metabolic, and immune etiologies (7). Among them, genetic factors play an important role in the pathogenesis of EEs. Many genes have been linked to EEs, and a significant proportion of them is ion channel genes which are particularly relevant to epilepsy (1, 8). Identifying the etiology of EEs is a primary clinical objective in the management of the disorder. Up to now, over 30 genes with precision medicine implications have been described in existing published literature (9, 10).

Sequencing-based studies have identified risk genes for rare and severe epilepsies and revealed a role of rare deleterious variation in common epilepsies (11). Whole exome sequencing (WES) is used routinely to detect sequence variants among the infants with EEs [single nucleotide variants (SNVs) and short insertions and deletions, SNPs/Indels] in clinical genetic laboratories (12, 13). However, the sequencing-based methods for identifying SNPs/Indels are not optimized for detecting the potential copy number variation (CNV), which is also involved in the pathogenesis of epilepsy, particularly early infantile epileptic encephalopathy (EIEE) (14, 15). This deficiency requires additional samples and application of genetic tests such as SNP arrays or array comparative genomic hybridization (aCGH) to discover the CNVs. Currently, many algorithms have been developed to obtain copy number information directly from the WES data (14, 16). This exome-based CNV analytic strategy has been proven to improve the diagnostic yields with cost-effective benefit (17). In this study, we evaluated the clinical application of profiling both SNPs/Indels and CNVs in a cohort of 73 infants with EEs who underwent WES as a diagnostic test.

## Methods

### Study Populations

A retrospective case study was performed in a single pediatric epilepsy center at Wuhan Children's Hospital, Tongji Medical College, Huazhong University of Science and Technology, Wuhan, China. The infants diagnosed with EEs were recruited between January 2018 and December 2019. All infants underwent a neurological examination, electromyography (EMG), and magnetic resonance imaging (MRI) for brain. And intellectual disabilities were assessed according to the criteria of the Diagnostic and Statistical Manual of Mental Disorders-5 (DSM-5) (18). All these clinical data were collected and independently reviewed by two neurologists (DS and YL).

### WES

Genomic DNA samples were extracted from peripheral blood using QIAamp^®^ Blood Mini Kit (Qiagen, Hilden, Germany). The quality of genomic DNA was evaluated by agarose gel electrophoresis analysis, and the quantity was measured by NanoDrop2000 and Qubit3.0. DNA was sheared with M220 Focused-ultrasonicator (Covaris, Woburn, MA, USA). DNA target regions were captured by hybridizing the genomic DNA sample library with the xGen^®^ Exome Research Panel v1.0 (IDT, USA). The captured and amplified DNA samples were sequenced using Illumina NovaSeq6000 (Illumina, San Diego, CA, USA) with 150 base-paired end reads.

### Detection of SNVs/Indels From WES Data

To identify disease-associated SNVs/Indels (short insertions and deletions smaller than 50 bp), sequencing data were analyzed according to an in-house pipeline. Both public software and commercial packages were implemented during bioinformatics analysis. Specifically, raw FASTQ was processed with FASTP (https://github.com/OpenGene/fastp) to cut adapters and filter out bad reads of low quality. The clean reads were then aligned against human reference (GRCh37) with BWA (19). SNPs/Indels were discovered by HaplotypeCaller tool of GATK after the necessary post-processes on primary alignment, including removal of duplicated reads, realignment, and base recalibrating (20). VEP(Ensembl Variant Effect Predictor) was employed to identify the effect of all discovered variants followed by variant annotation with AnnoVar (21). Notably, each variant was compared against several public databases, gnomAD, 1000 genomes project, NHLBI Exome Sequencing Project 6500 (ESP6500), and Exome Aggregation Consortium (ExAC) to achieve allele frequency in the general population. To identify the known and reported pathogenic variants, each variant was also compared against ClinVar (https://www.ncbi.nlm.nih.gov/clinvar) and Human Gene Mutation Database (HGMD) (www.hgmd.cf.ac.uk). In terms of possible influence on the protein function, variants were evaluated by several popular prediction tools, including InterVar, SIFT, PolyPhen, ClinPred, and GERP++ (22–26). Based on the variant annotations, series of filtering strategies were applied to identify candidate SNVs/Indels associated with EEs, which had been previously described (27).

### Detection of CNVs From WES Data

CNVs were detected by CNVkit (28). To create a stable and reliable CNV reference, in-house samples over the same sequencing protocol were selected for reference training in an iterative manner. Specifically, a set of 80 samples was used to create an initial CNV reference. CNV calling process was then run over this reference for each sample in the training set. Samples with any CNV event larger than 1 Mbp were excluded from the next iteration. In the next iteration, new samples were added in to make up the training set to be 80 samples. The iteration process was ended once no <50 samples were qualified. During the reference creation process, circular binary segmentation algorithm was chosen for CNV event segmentation, and the threshold parameter for copy number calling was set to be “−1.6, −0.8, 0.5, 1.” For better visualization investigation of CNV events, a tool was designed to plot copy number aligned with B-allele frequencies along the chromosome coordinates. The copy number was shown as log_2_ ratio obtained from bin level and segmented level CNV calling results. The B-allele frequencies were computed by samtools mpileup tool. AnnotSV and its annotation databases were locally installed to annotate detected CNV events for each tested sample for the following clinical interpretation (29).

### Confirmation and Interpretation of the Candidate Variants

To confirm the candidate SNVs/Indels, PCR amplification of the genomic DNA fragments of infants and their parents was performed, then the samples were sequenced by Sanger sequencing. The candidate CNVs identified were further verified by aCGH such as chromosomal microarray analysis (CMA) or methylation-specific multiplex ligation-dependent probe amplification (MS-MLPA). CMA was performed on Affymetrix GeneChip System 3000Dx v.2 (Thermo Fisher Scientific, MA, USA) by using CytoScan™ HD Array Kit (Thermo Fisher Scientific, MA, USA). Array data were analyzed with Affymetrix Chromosome Analysis Suite Software (Thermo Fisher Scientific, MA, USA). MS-MLPA was carried out using MS-MLPA Kit ME028 (MRC Holland, Amsterdam, Netherlands), and data analyses were performed using Coffalyser Software (MRC Holland, Amsterdam, Netherlands). The American College of Medical Genetics and Genomics/Association for Molecular Pathology (ACMG/AMP) criteria were applied in the interpretation of the pathogenicity of all identified variants (30).

### Statistical Analysis

Quantitative variables were expressed as median and interquartile range (IQR). Categorical variables were presented as numbers and percentages. Comparison of categorical variables was analyzed by Fisher's exact-test or chi-squared-test where appropriate. All statistical analyses were performed using SPSS software (version 23.0; SPSS Inc., Chicago, IL, USA).

## Result

### Demographic and Clinical Features of the Infants With EEs

Seventy-three infants with EEs admitted between January 2018 and February 2020 were enrolled in the single pediatric epilepsy center for the study. The median age was 7 months (range 0.03–12 months); 36 (49.32%) of them were males. Clinical features of the 73 infants are listed in Table 1. Thirty-two (43.84%) infants had been diagnosed with epilepsy syndrome. The most common type of syndrome was WS (22/73, 30.1%), followed by DS (20/77, 27.4%), OS (1/73, 1.4%), and MPEI (1/73, 1.4%). The remaining 29 patients (39.7%) were diagnosed as unclassified EIEE due to non-specific clinical manifestations. Fifty-four (73.97%) had intellectual development delay. Sixty-three (86.30%) infants had an abnormal record of EEG, and 25 (34.2%) had brain MRI abnormality. Focal seizure (31/73, 42.5%) was the most common onset feature, followed by tonic-clonic seizures (23/73, 31.5%) and tonic seizures (15/73, 20.5%).

**Table 1:** Demographic and baseline clinical characteristics of the patients with EEs.

**Demographics**	**Total (*****n*** **= 73)**		**Patients with P or LP variants (*****n*** **= 36)**		**Patients without P or LP variants (*****n*** **= 37)**		***P-*value**
	***n***	**%**	***n***	**%**	***n***	**%**	
Male sex	36	49.3	19	52.8	17	45.9	0.642
Intellectual/developmental disabilities	54	74.0	27	75.0	27	73.0	1.000
Multiple congenital anomalies	10	13.7	7	19.4	3	8.1	0.190
Abnormality in EEG	63	86.3					
Abnormality in brain MRI	25	34.2	15	41.6	10	27.0	0.223
**Epilepsy diagnosis**							
DS	20	27.4	11	30.6	9	24.3	0.607
WS	22	30.1	8	22.2	14	37.8	0.203
OS	1	1.4	1	2.7	0	0	0.493
MPEI	1	1.4	1	2.7	0	0	0.493
EIEE	29	39.7	15	41.6	14	37.8	0.813
**Epilepsy features**							
Focal seizures	31	42.5	20	55.6	11	29.7	0.034
Tonic seizures	15	20.5	5	13.8	10	27	0.247
Tonic-clonic seizures	23	31.5	10	27.8	13	35.1	0.616
Myoclonic seizures	4	5.5	1	2.78	3	8.1	0.615
Absence seizures	2	2.7	0	0	2	5.4	0.493
Infantile spasms	6	8.2	1	2.78	5	13.5	0.199

*DS, Dravet syndrome; WS, West syndrome; OS, ohtahara syndrome; MPEI, malignant migrating partial seizures; EIEE, early infantile epileptic encephalopathy; P, pathogenic; LP, likely pathogenic*.

### Identification of Pathogenic SNVs/Indels

For each sample, an average of 43.9 M (±5.9 M) pairs of 150 bp raw reads of WES were obtained. The exonic target region capture efficiency was 84.5% (±2.5%) on average. The mean sequencing depth on exonic target regions with flanking 10 bp on either side was 151-folds (±20-folds), providing a fairly high coverage of targeted exonic regions. Such high average coverage also ensured the detection of CNVs according to the demand of CVNkit. On average, 80,753 variants were called in each sample, consisting of 72,522 SNVs and 8,231 Indels. After excluding low quality variants with depths lower than 20-folds, about 68,741 SNVs and 7,382 Indels were left per sample.

When interpreted by ACMG recommended standards and analyzed for clinical concordance, 30 SNVs/Indels in 29 infants (39.7%) were graded pathogenic or likely pathogenic variants associated with EEs (Table 2). Among these variants, 15 (50.0%) were missenses, and 15 (50.0%) were protein-truncating variants including 4 (13.3%) non-senses, 10 (33.3%) frameshift Indels, and 1 (3.3%) splicing site variant. The majority of the variants were inherited in *de novo* pattern (20, 66.7%), defined as variants present in the infants but not in the parents. Two variants (6.7%) were inherited in X-linked pattern, and one variant (3.3%) in the *PCDH19* gene was inherited in male-sparing pattern. Moreover, 16 (53.3%) variants were previously unreported in variant databases.

**Table 2:** Profiles of identified pathogenic or likely pathogenic sequence variants in the patients with EEs.

**ID**	**Age (months)**	**Gender**	**Clinical phenotype**	**Coordinates (GRCh37/hg19)**	**Ref**	**Gene**	**Nucleotide substitution**	**Amino acid substitution**	**Parental origin**	**Interpretation**	**Novel/reported**
EE2	3	M	DS	chr2:166900484	NM_001165963	SCN1A	c.1738C>T	p.R580X	*De novo*	P	Reported
EE5	8	M	EIEE	chr1:224619250-224619256	NM_001115113	WDR26	c.550_556delCCTTTAG	p.P184Cfs*9	*De novo*	P	Novel
EE8	2	F	EIEE	chr1:97915746	NM_000110	DPYD	c.1774C>T	p.R592W	Mother	LP	Reported
				chr1:97547896	NM_000110	DPYD	c.2897C>T	p.S966F	Father	LP	Novel
EE7	4	M	DS	chr2:166896016	NM_001165963	SCN1A	c.2506G>T	p.D836Y	*De novo*	LP	Novel
EE9	6	F	DS	chr2:166866296	NM_001165963	SCN1A	c.3934dupA	p.I1312fs	Father	LP	Novel
EE11	7	M	DS	chr2:166870339	NM_001165963	SCN1A	c.3620T>C	p.L1207P	*De novo*	LP	Reported
EE12	6	F	DS	chr2:166896032	NM_001165963	SCN1A	c.2490delA	p.G831Afs*10	NA	LP	Novel
EE14	8	M	WS	chr9:101216454	NM_005458	GABBR2	c.1045G>A	p.V349M	NA	LP	Reported
EE18	8	M	WS	chr1:181745289	NM_001205294	CACNA1E	c.5135G>A	p.R1712Q	*De novo*	LP	Novel
EE20	5	F	DS	chr2:166848251-166848254	NM_001165963	SCN1A	c.5531_5534delTTTG	p.P1844fs	*De novo*	P	Novel
EE21	12	F	EIEE	chr2:166237178	NM_001040143	SCN2A	c.4385delT	p.F1462fs	*De novo*	P	Novel
EE23	6	F	EIEE	chr2:166848621	NM_001165963	SCN1A	c.5164A>G	p.T1722A	*De novo*	LP	Reported
EE32	9	M	EIEE	chr2:166898844	NM_001165963	SCN1A	c.2050C>T	p.R684X	*De novo*	P	Reported
EE33	12	F	EIEE	chrX:99657797	NM_001184880	PCDH19	c.2341delA	p.I781fs	*De novo*	P	Reported
EE39	2	F	MPEI	chr9:138660694	NM_020822	KCNT1	c.1421G>A	p.R474H	Mother	LP	Reported
EE40	1	F	OS	chr20:62070961	NM_004518	KCNQ2	c.917C>T	p.A306V	NA	LP	Reported
EE42	12	M	DS	chr2:166892659-166892660	NM_001165963	SCN1A	c.3327_3328delTG	p.P1109fs	*De novo*	P	Novel
EE43	11	M	EIEE	chr2:166908355	NM_001165963	SCN1A	c.837dupA	p.W280fs	NA	LP	Novel
EE46	1	M	EIEE	chr2:166245951	NM_001040143	SCN2A	c.5635A>G	p.M1879V	*De novo*	LP	Novel
EE47	3	F	EIEE	chr2:166898801	NM_001165964	SCN1A	c.2092+1G>T	NA	*De novo*	P	Novel
EE48	4	F	EIEE	chr17:29588751	NM_000267	NF1	c.4537C>T	p.R1513X	Father	P	Reported
EE49	1	M	EIEE	chr20:62071057	NM_004518	KCNQ2	c.821C>T	p.T274M	*De novo*	P	Reported
EE51	6	M	EIEE	chr2:166019317	NM_001081676	SCN3A	c.716C>A	p.A239D	*De novo*	LP	Novel
EE60	7	F	EIEE	chr12:52082568	NM_001177984	SCN8A	c.641G>A	p.G214D	*De novo*	P	Reported
EE61	2	M	EIEE	chrX:18646629-18646630	NM_001323289	CDKL5	c.2635_2636delCT	p.L879fs	Mother	P	Novel
EE63	3	F	WS	chr5:161317979	NM_000806	GABRA1	c.779C>T	p.P260L	*De novo*	P	Reported
EE65	6	F	EIEE	chr12:52159789	NM_001177984	SCN8A	c.2879T>A	p.V960D	*De novo*	P	Reported
EE66	6	M	DS	chr2:166908376	NM_001202435	SCN1A	c.817C>G	p.L273V	*De novo*	LP	Novel
EE70	3	M	WS	chr9:138660693	NM_020822	KCNT1	c.1420C>T	p.R474C	*De novo*	LP	Reported

*DS, Dravet syndrome; WS, West syndrome; OS, ohtahara syndrome; MPEI, malignant migrating partial seizures; EIEE, early infantile epileptic encephalopathy; P, pathogenic; LP, likely pathogenic*.

These pathogenic or likely pathogenic SNVs/Indels were located in 14 different genes. The genes, encoding sodium and potassium channels of neuron and maintaining neuron excitability, were the prevalent disease genes and account for 22 (30.1%) infants with EEs. Variants of the *SCN1A* gene, which encodes sodium voltage-gated channel alpha subunit 1, accounted for the largest proportion (12/73, 16.4%) of positively detected infants. And variants of the other ion channels genes, *SCN2A, SCN8A, KCNQ2*, and *KCNT1*, accounted for 2.7% (2/73), respectively. One pathogenic variant in *de novo* status was found in the *CACNA1E* gene, which encodes calcium voltage-gated channel subunit alpha1 E. The disease-causing variants in *CACNA1E* has recently been identified as a cause of developmental and epileptic encephalopathies (31). One variant each was found in both *GABRA1* and *GABRA2* genes, encoding Gamma-aminobutyric acid (GABA) type A receptor subunits. GABA type A receptors are pentameric chloride channels, which are the principal receptors that mediate the inhibitory synaptic transmission in the mammalian brain. Mutations in the genes encoding the GABA receptor subunits have been associated with a spectrum of neurological disorders including EEs. From the current study, four variants were located in the epilepsy-related genes, the *DPYD* gene, *NF1* gene, and *WDR26* gene. Two compound heterozygous variants were found in the *DPYD* gene, which encodes the rate-limiting enzyme for fluoropyrimidine catabolism and eliminates over 80% of administered 5-FU (32). The pathogenic homozygous or compound heterozygous variants within the *DPYD* gene are associated with dihydropyrimidine dehydrogenase (DPD) deficiency, and DPD-deficient infants may develop intellectual disability, motor retardation, and seizures. One non-sense variant was detected within the *NF1* gene in the infant with WS. The *NF1* gene is a tumor suppressor gene; therefore, loss of function due to a mutation leads to increase in cell proliferation and results in the development of neurofibromatosis type 1. Apart from tumors, patients with neurofibromatosis type 1 have an increased risk for WS (33). One novel *de novo* fame-shift variant was identified in the *WDR26* gene, and *WDR26* haploinsufficiency has been linked to a syndrome characterized by intellectual disability, seizures, abnormal gait, and distinctive facial features.

There were 21 candidate variants in 15 infants (20.5%) that were evaluated to be variants of uncertain significance (VUS) by ACMG/AMP criteria (Supplementary Table 1). These variants were kept through filtering steps because of pathogenicity prediction inconsistency in multiple perdition tools and limited published literature information.

### Identification of Pathogenic CNVs

**Figure 1:**
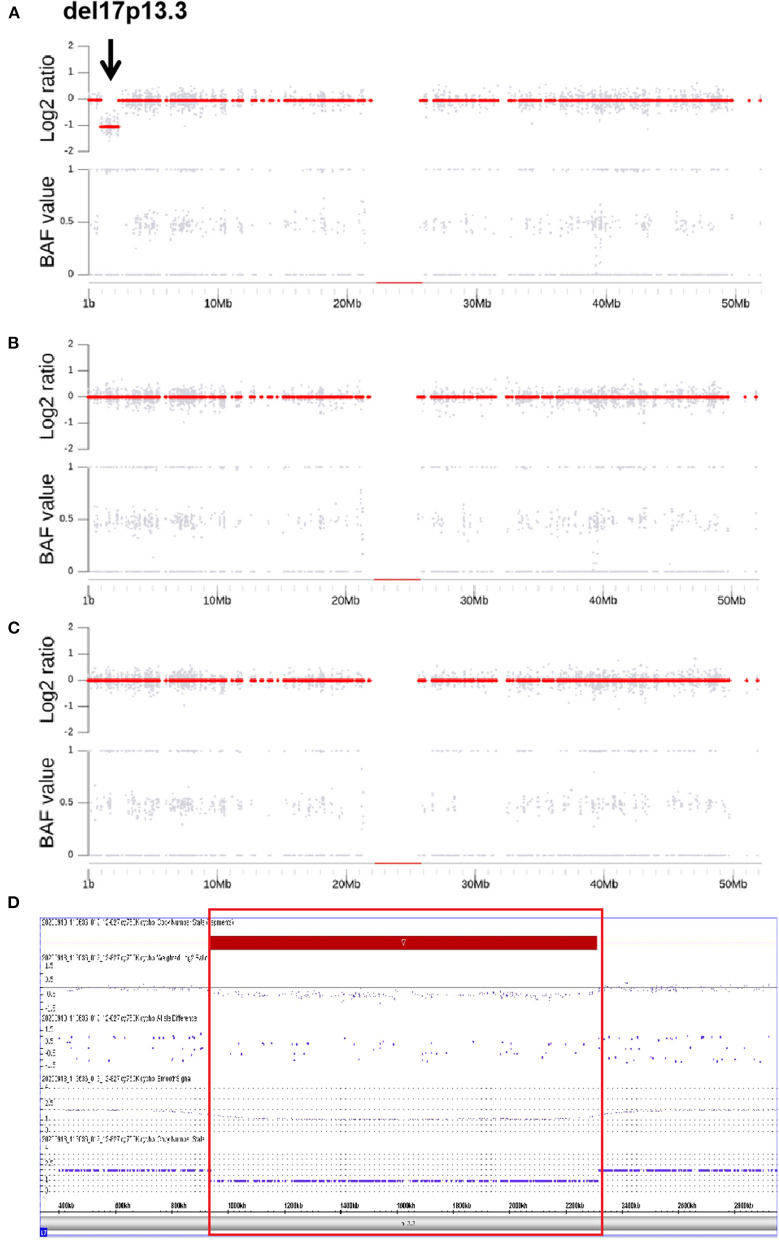
B allele Frequency, Log_2_ Ratio plots and CMA of a *de novo* heterozygous deletion of 17p13.3 in the patient with Miller-Dieker syndrome. In **(A–C)**, the upper and the lower subplots are the copy number estimation and the B allele Frequency (BAF, allelic copy ratio) in the relevant regions. The copy number estimation is provided as log_2_ ratio (the upper subplots) which is calculated by log_2_ (*N*_*e*_/*N*_*d*_), where *N*_*e*_ is the estimated copy number and *N*_*d*_ is the normal copy number of the diploid (i.e., 2 for autosome). The gray dots show the Log_2_ Ratios of the bins and the red dots are the ones of the segments. **(A–C)** are the copy number Log_2_ Ratio and BAF plots for the proband, father and mother, respectively. The proband **(A)**, the values of Log_2_ Ratio in detected region are around −1 and being recognized as a segment (red dots of −1). Meanwhile, the BAF are all closing to 0 or 1, indicating the homozygosity of this segment. A deletion of 17p13.3 is indicated by BAF band along with the lack of change in Log_2_ Ratio (black arrow, **A**). Trio analysis confirms that the deletion has occurred *de novo* in the proband. In **(D)**, the deletion was verified by MS-MLPA and the loss in 17p13.3 is indicated by red rectangle.

Through CNV analysis, six CNVs were linked to the clinical phenotype among six cases (8.2%) (Table 2). All of these variants were in *de novo* status. Five discovered CNVs were deletions of 2q24.3, 1p36, 15q11–q13, 16p11.2, and 17p13.3. Patient 73, who was a 10-month-old female, was admitted for infantile spasms, and brain MRI disclosed the lissencephaly. Both the CNV analysis and CMA found a *de novo* heterozygous deletion of 17p13.3 which was indicated by a value log_2_ ratio = −1 and missing value of 0.5 in BAF (Figure 1). In the deletion region, the *YWHAE* and *CRK* genes on the telomeric end of chromosome 17p have been considered as epilepsy-causing genes in previous clinical reports, and the deletion was interpreted as pathogenic (34, 35). The clinical findings and genetic test supported the diagnosis of Miller–Dieker syndrome. All these five deletions were classified as pathogenic, which included genes that are well-known to cause epilepsy (Table 2).

One large duplication of 4q11–q13.1 was detected in a 12-month-old female with generalized tonic-clonic seizures and growth retardation. A previous study had detected duplication at 4q11–q13.1 in patients with idiopathic short stature, and *TMEM165* and *POLR2B* in this region were suspected as candidate genes for growth retardation (36, 37). The duplication also included the *SRD5A3* gene, the cause of Kahriz syndrome, and the null mutation of *SRD5A3* having been reported to be associated with the epileptic phenotype, WS (38). In the duplicated regions, we also considered the REST gene as another candidate disease-causing gene because of its potential to control fundamental transcription patterns that drive circuit excitability, seizures, and epilepsy (39). However, due to the large size of duplication, it was difficult to determine the disease-causing gene(s), and the duplication was interpreted as VUS.

Altogether, the genetic causes (pathogenic or likely pathogenic variants) of EEs were successfully discovered for 34 (46.6%) infants. Twenty-nine (39.7%) infants carried the SNVs/Indels, and five (6.8%) carried CNVs related to the EEs. And the majority of the disease-causing variants were inherited in *de novo* pattern (25, 71.4%).

### Genotype to Phenotype Correlation

In 20 clinical cases of DS, half of them (10/20, 50%) were found to arise from pathogenic or likely pathogenic *SCN1A* variants, and none of the disease-causing variants were identified in the other genes. In contrast, in 22 infants with WS, 8 cases (7/22, 31.8%) were caused by pathogenic or likely pathogenic variants from a variety of genes, including *GABRA1, GABRA2, KCNT1, SCN3A, CACNA1E, PHGDH*, and *PRRT2* (in del 16p11.2). One infant was diagnosed with OS, and a likely pathogenic variant was found in *KCNQ2*. Additionally, another one infant was diagnosed with MPEI, and a likely pathogenic variant was found in *KCNT1*. As shown in Table 3, three pathogenic CNVs del1p36, del15q11–q13, and del17p13.3 corresponded to 1p36 deletion syndrome, Angelman syndrome, and Miller–Dieker syndrome, respectively.

**Table 3:** Profiles of identified rare CNVs in the patients with EEs.

**ID**	**Age at onset (m)**	**Gender**	**Cytoband**	**Clinical diagnosis**	**Coordinates (GRCh37/hg19)**	**CNV**	**Size**	**Relevant genes**	**Parental origin**	**Interpretation**	**Novel/reported**
EE22	8	M	1p36	1p36 deletion syndrome	chr1:736,369-5660,269	Del	4.92 Mb	*GABRD, GNB1, SKI*	*De novo*	P	Reported
EE27	12	F	4q11-q13.1	NA	chr4:52,685,118-65,914,334	Dup	13.23Mb	*SRD5A3, REST*	*De novo*	VUS	Novel
EE38	6	F	2q24.3	Dravet syndrome	chr2:166858942-166850970	Del	7.972kb	*SCN1A*	*De novo*	P	Novel
EE58	10	F	15q11-q13	Angelman syndrome	chr15:22471433-29444050	Del	6.972Mb	*UBE3A,GABRB3*	*De novo*	P	Reported
EE72	3	M	16p11.2	16p11.2 deletion syndrome	chr16:29,675,049-30,199,897	Del	524.848Kb	*PRRT2*	*De novo*	P	Reported
EE73	10	F	17p13.3	Miller-Dieker syndrome	chr17:934,739-2,304,030	Del	1.369Mb	*YWHAE, CRK*	*De novo*	P	Reported

*P, pathogenic variant; VUS, a variant of uncertain significance*.

## Discussion

In the study, 73 infants with EEs for both SNPs/Indels and CNVs were evaluated by analyzing the WES data generated from a commercial human exome panel, and 34 mutations were found. SNVs/Indels accounted for 39.7% cases, and the detection of CNVs by using CNVkit increased a diagnostic rate of 6.8%. This is the first report of detecting both SNPs/Indels and CNVs in a single test for genetic diagnosis of EEs.

Through the current study, pathogenic and likely pathogenic SNPs/Indels were identified in 14 different disease-causing genes, and pathogenic CNVs were associated with at least 9 potential disease-causing genes. Altogether, 23 genes were involved in the pathogenesis of EEs in the current cohort. Similar to previous studies, ion channel-related genes, especially sodium ion channels and potassium ion channels, accounted for the large proportion of pathogenic and likely pathogenic variants. Indeed, in half of these genes, variants have been recorded to produce truncating proteins (40). One novel pathogenic variant (c.5135G>A/p.R1712Q) in *de novo* status was identified in a calcium channel-related gene, *CACNA1E*, which encodes a functionally critical subunit of a high voltage-activated, rapidly inactivating R-type calcium channel and initiates rapid synaptic transmission in the brain. The infant harboring the variant had intellectual disability and hypsarrhythmia on EEG, consistent with a diagnosis of WS. Helbig et al. had identified *de novo CACNA1E* variants in 30 individuals with EEs, characterized by refractory infantile-onset seizures, severe hypotonia, and profound developmental impairment (31). Most of the pathogenic variant clusters within the cytoplasmic ends of all the four S6 segments (Domains I, II, III, and IV) form the presumed CaV2.3 channel activation gate. The p.R1712Q variants also located in the S6 segment (Domain IV) are supposed to play a gain-of-function effect comprising facilitated voltage-dependent activation and slowed inactivation (31). One novel fame-shift deletion (c.550_556delCCTTTAG/p.P184Cfs^*^9) in the *WDR26* gene was detected in an infant with absence seizures and febrile seizures, ventriculomegaly, intellectual/developmental disabilities, congenital heart disease, facial dysmorphism, and yellow hair. As WDR26 is located in chromosomal region 1q42, this showed that the patient shared some clinical features with 1q41q42 microdeletion syndrome, such as seizures, intellectual/developmental disabilities, and facial dysmorphism (41). Therefore, the truncation was supposed to lead to a haploinsufficiency of *WDR26* to cause EEs like pathogenic mechanism of 1q41q42 microdeletion (41).

The majority of positive cases with EEs resulted to *de novo* pathogenic or likely pathogenic variants. Twenty SNPs/Indels and five CNVs in *de novo* status accounted for 71.4% positive findings and explained about one third of the infants with EEs overall. However, the current study was unable to exclude the possible parental mosaicism, which remains undetected by current methods. Parental mosaicism had been shown to present significantly higher recurrence risk in future pregnancies than the apparent *de novo* variants. It was identified in ~10% of families with children with an apparent *de novo SCN1A* variant in EEs (42). For further genetic counseling with apparent *de novo* variants in EEs, there is a need to consider higher recurrence risk especially for families who may wish to have another baby, who should therefore be subjected to more tests to confirm the status of *de novo*.

In our cohort, WS showed great heterogeneity in genetic etiology, with seven genes accounting for seven cases. And both SNP/Indels or CNVs were identified as causatives in WS. Currently, the routine treatment includes adrenocorticotropic hormone, vigabatrin, and corticosteroids. However, with the understanding of disease-causing genes and related pathogenesis, new therapies that target specific pathways of pathogenesis to correct the underlying molecular dysfunction will be developed in the near future. For example, there are a number of trials including rapamycin/everolimus, targeting the mTOR pathway, for the treatment of tuberous sclerosis complex and related WS (43); retigabine for the treatment of *KCNQ2*-related WS (44); and topiramate for the treatment of *CACNA1E*-related WS (31).

The detection of CNVs by using CNVkit increased the diagnostic rate by 6.8% in our cohort. The traditional approach for detecting genome-wide CNVs includes karyotyping, fluorescence *in situ* hybridization (FISH), SNP array, and aCGH. Among them, aCGH has been proven as an indispensable approach to screen CNVs associated with epilepsy, intellectual disability, developmental delay, or congenital anomalies in children (45). In the past decade, WES, which refers to sequencing of all protein-coding exons, has been widely used in genetic testing in Mendelian disorders as well as other complex diseases (46). It is powerful to detect SNVs/Indels in enriched protein-coding regions. Recently, many bioinformatics tools have been developed to do CNVs calling from WES data. These included XHMM (47), using hidden Markov model, and CoNIFER (48), using singular value decomposition, whereas others such as cn.MOPS use Bayesian inference (49). CNVkit is a toolkit that uses both the targeted reads and the non-specifically captured off-target reads to achieve highly accurate and reliable copy ratio estimates across the genome (28). All these CNV calling tools based on WES data could be considered as a complementary way with only computational effort to improve the diagnostic yields in EEs. In our study, aCGH served as a confirmatory tool rather than a screening tool and could bring cost-effective benefit for clinical diagnosis.

There are various limitations of the current study that need to be addressed. First, phenotypic details for the parents and familial disease history were lacking in some cases, which is a common issue with retrospective studies. Second, the current study approach was to identify known reported disease-causing genes and their rare variants and cloud not identify the variants in new disease-causing genes and common variants as genetic risks for EEs. Third, there were 22 variants (21 SNPs/Indels and 1 CNV) in 16 infants that were interpreted as VUS in EEs. Most of them were novel findings when they were compared with disease variants database or published literature. As more evidence emerges through function analysis, some VUS will be reclassified as the pathogenic variants. All these limitations may influence genetic diagnostic rate under the current study.

In summary, the application of both SNPs/Indels and CNVs detection in a single test based on WES yielded diagnosis in 46.6% of the infants with EEs, which demonstrates the utility of this pipeline as a diagnostic test for pediatric patients with a disease presenting a highly phenotypic and genetic heterogeneity. Importantly, a number of novel variants involved in EEs are being reported here. Although there are technical challenges with CNVs calling as well as challenges for pathogenic classification of identified variants, WES may serve as a first-tier test with cost-effective benefit in EEs.

## Data Availability Statement

The raw sequence data reported in this paper have been deposited in the Genome Sequence Archive in National Genomics Data Center, China National Center for Bioinformation/Beijing Institute of Genomics, Chinese Academy of Sciences (https://bigd.big.ac.cn/). After publication of study findings, the data will be available for others to request. The research team will provide accession number of the database once the data are approved to be shared with others. The proposal with description of study objectives will be needed for evaluation of the reasonability to request for the data. The corresponding authors and the Ethical Committee of Wuhan Children's Hospital will make a decision based on these materials.

## Ethics Statement

The studies involving human participants were reviewed and approved by The Ethical Committee of Wuhan Children's Hospital, Tongji Medical College, Huazhong University of Science and Technology, Wuhan, China. Written informed consent to participate in this study was provided by the participants' legal guardian/next of kin.

## Author Contributions

FZ and DS conceived, supervised this study, and wrote the manuscript. DS, YaL, WC, and ZL evaluated and cared for the patients. JM, KN, and MC collected clinical data. CW and YZ performed the WES test. FZ and YoL performed the bioinformatics analysis of WES data. All authors contributed to the article and approved the submitted version.

## Conflict of Interest

YoL and YZ are employees of company Aegicare Technology Co., Ltd. (Shenzhen, China). The remaining authors declare that the research was conducted in the absence of any commercial or financial relationships that could be construed as a potential conflict of interest.
