# Supplementary material for: Detection of Disease-Causing SNVs/Indels and CNVs in Single Test Based on Whole Exome Sequencing: A Retrospective Case Study in Epileptic Encephalopathies
Source: Front Pediatr. 2021 May 13;9:635703. doi: 10.3389/fped.2021.635703 (PMC8155357; doi:10.3389/fped.2021.635703)
Supplement: Supplementary file 1 [file Table_1.DOCX]

| **ID** | **Age** | **gender** | **Clinical**  **Phenotype** | **chr** | **positions** | **NM-ID** | **gene** | **nucleotide substitution** | **Amino acid substitution** | **Parental origin** | **interpretation** | Novel/ reported |
| --- | --- | --- | --- | --- | --- | --- | --- | --- | --- | --- | --- | --- |
| EE9 | 6m | F | DS | 2 | 166866300 | NM_001165963 | SCN1A | c.3931G>A | p.A1311T | NA | VUS | Novel |
| EE10 | 12m | M | WS | 2 | 166170254 | NM_001040143 | SCN2A | c.1159G>A | p.E387K | Mother | VUS | Novel |
| EE15 | 2m | F | EIEE | 5 | 45695992-45695994 | NM_021072 | HCN1 | c.202_204del | p.68_68del | NA | VUS | Novel |
|  |  |  |  | 5 | 113698645 | NM_021614 | KCNN2 | c.173_174insCGC | p.Ala59dup | NA | VUS | Novel |
| EE24 | 9m | M | Dravet | 5 | 45267295 | NM_021072 | HCN1 | c.1679G>A | p.R560H | Father | VUS | Novel |
| EE25 | 5m | M | WS | 1 | 120286583 | NM_006623 | PHGDH | c.G1522A | p.V508I | NA | VUS | Novel |
|  |  |  |  |  | 120278017 | NM_006623 | PHGDH | c.743C>T | p.A248V | NA | LP | Novel |
| EE26 | 12m | M | DS | 2 | 166848674 | NM_001165963 | SCN1A | c.5111T>C | p.M1704T | Mother | VUS | Novel |
| EE31 | 10m | M | EIEE | 2 | 166850721 | NM_001165963 | SCN1A | c.4703G>A | p.R1568H | Father | VUS | Reported |
| EE35 | 12m | F | EIEE | 7 | 66103259 | NM_001167961 | KCTD7 | c.334C>G | p.R112G | Father | VUS | Novel |
|  |  |  |  |  | 66104035 | NM_001167961 | KCTD7 | c.686A>T | p.D229V | Mother | VUS | Novel |
| EE36 | 12m | M | EIEE | 19 | 35524653 | NM_199037 | SCN1B | c.458G>A | p.G153D | NA | VUS | Novel |
|  |  |  |  | 20 | 47991283 | NM_004975 | KCNB1 | c.814C>T | p.P272S | NA | VUS | Novel |
| EE37 | 9m | F | Dravet | 3 | 47061265 | NM_014159 | SETD2 | c.7416A>C | p.E2472D | Father | VUS | Novel |
|  |  |  |  |  | 47163843 | NM_014159 | SETD2 | c.2283G>A | p.M761I | Mother | VUS | Reported |
| EE45 | 12m | F | EIEE | 15 | 93522431 | NM_001271 | CHD2 | c.2794A>G | p.K932E | NA | VUS | Novel |
| EE50 | 12m | F | EIEE | 16 | 1248684 | NM_001005407 | CACNA1H | c.713T>G | p.F238C | NA | VUS | Novel |
| EE52 | 2m | M | EIEE | 20 | 62065252 | NM_004518 | KCNQ2 | c.1028C>T | p.A343V | NA | VUS | Novel |
| EE53 | 12m | M | EIEE | 5 | 89990278 | NM_032119 | ADGRV1 | c.7705G>A | p.V2569I | NA | VUS | Novel |
|  |  |  |  |  | 90055367 | NM_032119 | ADGRV1 | c.12082G>A | p.D4028N | NA | VUS | Novel |
| EE57 | 8m | F | EIEE | 11 | 35282496 | NM_001252652 | SLC1A2 | c.1643A>G | p.N548S | Mother | VUS | Novel |

**Supplementary table 1. Profiles of identified VUS in the patients with EEs**

DS Dravet syndrome; WS West syndrome;OS ohtahara syndrom; EIEE early infantile epileptic encephalopathy;LP likely pathogenic; VUS uncertain significance.
